# Retraction for Megahed et al., “Complete Genome Sequences of Four Major Viruses Infecting Marine Shrimp in Egypt”

**DOI:** 10.1128/MRA.01266-19

**Published:** 2019-10-31

**Authors:** Mohamed E. Megahed, Roberto Cruz-Flores, Arun K. Dhar

**Affiliations:** a National Institute of Oceanography and Fisheries (NIOF), Gulfs of Suez & Aqaba’s Branch, Attaka, Suez, Egypt; b Aquaculture Pathology Laboratory, School of Animal and Comparative Biomedical Sciences, The University of Arizona, Tucson, Arizona, USA

## RETRACTION

Volume 7, no. 9, e00809-18, 2018, https://doi.org/10.1128/MRA.00809-18. We retract this article. The sequences were submitted to the NCBI GenBank (accession no. KT316249 to KT316254 and KT316256 to KT316260 on 15 December 2015 for PstDNV1, KR492908 to KR492911 on 27 October 2015 and KT316240 to KT316245 on 15 December 2015 for HPV, KT316279 on 15 December 2015 for GAV, and KT316278 on 15 December 2015 for YHV) by the primary author, Mohamed E. Megahed, National Institute of Oceanography and Fisheries (NIOF), Suez, Egypt. Unfortunately Dr. Megahed has been instructed by NIOF, Suez, Egypt, not to share the raw sequence data under the Law No. 82/2002 and via a declaration of the Deputy Minister of Agriculture & Land Reclamation. Due to this inability to share the raw sequence data, we are retracting the article.

